# Metabolic Profiling of Alternative NAD Biosynthetic Routes in Mouse Tissues

**DOI:** 10.1371/journal.pone.0113939

**Published:** 2014-11-25

**Authors:** Valerio Mori, Adolfo Amici, Francesca Mazzola, Michele Di Stefano, Laura Conforti, Giulio Magni, Silverio Ruggieri, Nadia Raffaelli, Giuseppe Orsomando

**Affiliations:** 1 Department of Clinical Sciences, Section of Biochemistry, Polytechnic University of Marche, Ancona, Italy; 2 School of Life Sciences, University of Nottingham, Medical School, Queen's Medical Centre, Nottingham, United Kingdom; 3 School of Biosciences and Biotechnology, University of Camerino, Camerino, Italy; 4 Department of Agricultural, Food and Environmental Sciences, Polytechnic University of Marche, Ancona, Italy; University of Florida, United States of America

## Abstract

NAD plays essential redox and non-redox roles in cell biology. In mammals, its *de novo* and recycling biosynthetic pathways encompass two independent branches, the “amidated” and “deamidated” routes. Here we focused on the indispensable enzymes gating these two routes, *i.e.* nicotinamide mononucleotide adenylyltransferase (NMNAT), which in mammals comprises three distinct isozymes, and NAD synthetase (NADS). First, we measured the *in vitro* activity of the enzymes, and the levels of all their substrates and products in a number of tissues from the C57BL/6 mouse. Second, from these data, we derived *in vivo* estimates of enzymes'rates and quantitative contributions to NAD homeostasis. The NMNAT activity, mainly represented by nuclear NMNAT1, appears to be high and nonrate-limiting in all examined tissues, except in blood. The NADS activity, however, appears rate-limiting in lung and skeletal muscle, where its undetectable levels parallel a relative accumulation of the enzyme's substrate NaAD (nicotinic acid adenine dinucleotide). In all tissues, the amidated NAD route was predominant, displaying highest rates in liver and kidney, and lowest in blood. In contrast, the minor deamidated route showed higher relative proportions in blood and small intestine, and higher absolute values in liver and small intestine. Such results provide the first comprehensive picture of the balance of the two alternative NAD biosynthetic routes in different mammalian tissues under physiological conditions. This fills a gap in the current knowledge of NAD biosynthesis, and provides a crucial information for the study of NAD metabolism and its role in disease.

## Introduction

NAD is pivotal for cell life, first as a reusable redox coenzyme for energy production, second as a consumable substrate in enzymatic reactions regulating crucial biological processes, including gene expression, DNA repair, cell death and lifespan, calcium signaling, glucose homeostasis, and circadian rhythms [1]–[5]. In this view, the key role played by NAD-consuming enzymes in cell biology and pathophysiology has recently renewed an emerging interest on NAD regeneration and its metabolic regulation [6], [7], so that NAD biosynthesis increasingly appears a promising target for the treatment of a number of human diseases [4], [8]–[12].

**Figure 1:**
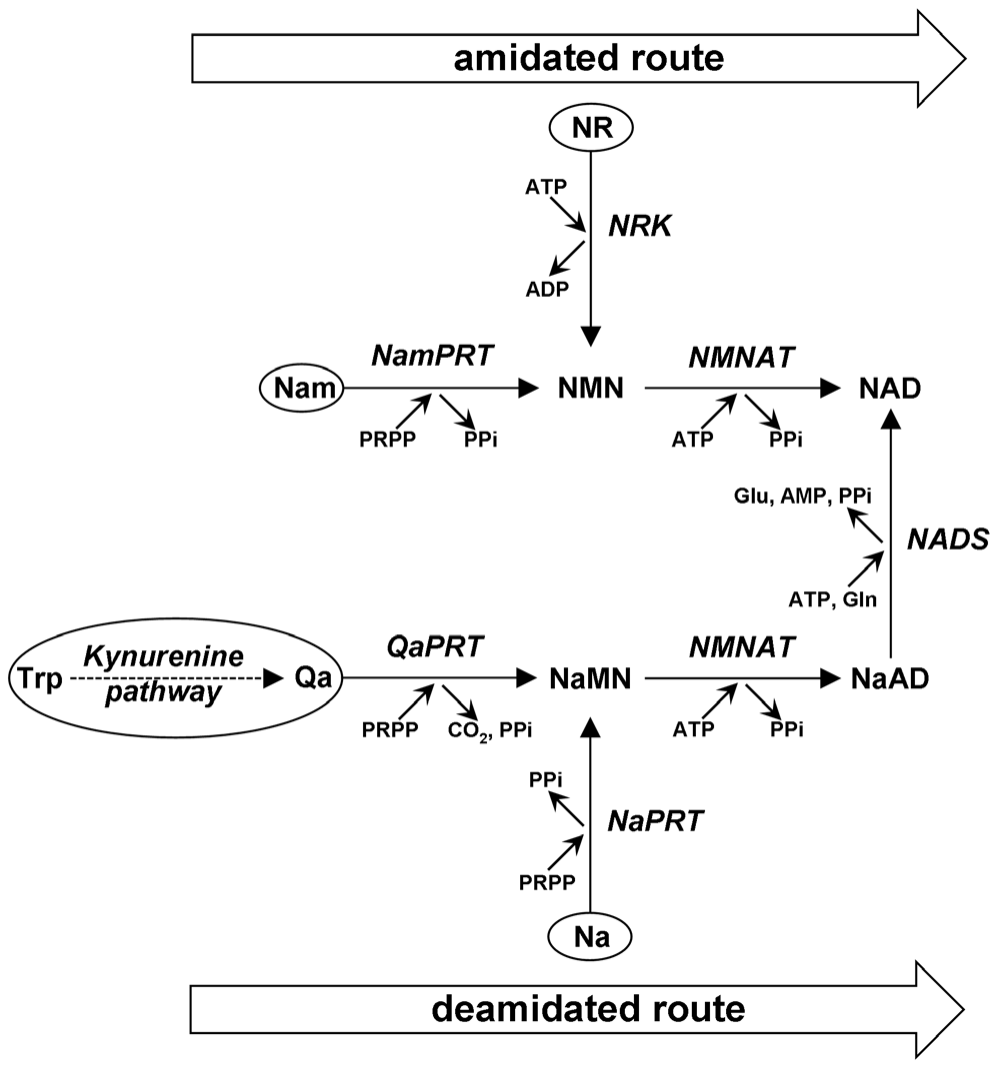
Metabolic pathway of NAD biosynthesis in mammals. The two routes, “amidated” and “deamidated”, are highlighted. The different sources of the pyridine moiety for NAD synthesis are circled. The metabolites involved are: nicotinic acid (Na), nicotinamide (Nam), nicotinamide riboside (NR), quinolinic acid (Qa), nicotinate mononucleotide (NaMN), nicotinamide mononucleotide (NMN), nicotinate adenine dinucleotide (NaAD), and nicotinamide adenine dinucleotide (NAD). The enzymes involved are: QaPRT (EC 2.4.2.19), NaPRT (EC 2.4.2.11), NamPRT (EC 2.4.2.12), NRK (EC 2.7.1.22), NMNAT (EC 2.7.7.1), and NADS (EC 6.3.5.1).

The NAD biosynthetic pathway operating in mammals [11]–[13], as summarized in Figure 1, is composed by a *de novo* route starting from tryptophan, and three alternative recycling routes starting from pre-formed pyridine moieties, *i.e.* nicotinic acid (Na), nicotinamide (Nam), and nicotinamide riboside (NR). These NAD precursors, collectively referred to as niacin or vitamin B3 [6], may arise from dietary supply and/or intracellular NAD catabolism. A key *de novo* intermediate is quinolinic acid (Qa), the end product of the kynurenine pathway, further converted by three subsequent steps into nicotinate mononucleotide (NaMN), nicotinate adenine dinucleotide (NaAD), and NAD. NaMN can also be synthesized directly from Na; the deamidated recycling route from Na to NAD is frequently referred to as the Preiss-Handler pathway [14]. Two amidated recycling routes are also operative, whereby either Nam or NR are first converted to nicotinamide mononucleotide (NMN), and then to NAD. Thus, NAD biosynthesis in mammals may occur via distinct “amidated” and “deamidated” routes, that connect to each other only after the dinucleotide NaAD has been formed, and show no other cross-talk at any intermediate level (Figure 1).

This dual pathway involves several enzymes forming the pyridine mononucleotides, and two enzymes forming the pyridine dinucleotides. The latter, catalyzing reactions common to both NAD recycling and *de novo* synthesis, are nicotinamide mononucleotide adenylyltransferase (NMNAT, EC 2.7.7.1) and, NAD synthetase (NADS, EC 6.3.5.1). In mammals, NMNAT is represented by three distinct gene products, a nuclear homoexameric NMNAT1 isozyme, a cytosolic monomeric NMNAT2 isozyme, and a mitochondrial homotetrameric NMNAT3 isozyme [15]–[17]. They display differential properties *in vitro* [18], [19], but catalyze the same ATP-dependent adenylation reaction *in vivo* using either NMN or NaMN as substrates, to yield the corresponding dinucleotides and PPi. Therefore, they operate equally in the “amidated” and “deamidated” NAD biosynthetic routes (Figure 1). Conversely, mammalian NADS is known as a homohexameric cytosolic protein encoded by a single gene. It catalyzes an amidation reaction converting NaAD to NAD, using both ATP and glutamine as co-substrates with concomitant production of AMP, PPi, and glutamic acid [20]. The essential role of both NMNAT and NADS has been confirmed by gene deletion and knocking down experiments in different organisms [21]–[24]. While no evidence has been so far reported on the involvement of NADS in pathological conditions, NMNAT gene alterations have been recently linked to cancer [25], Leber's congenital amaurosis [26], and axon protection in several neurodegeneration and acute injury models, including Wallerian degeneration models [27].

Recent knowledge on the physiological distribution in mammalian tissues of the NAD-related metabolites has been steadily growing thanks to the progress of analytical detection methods [28]–[30]. Evidence about the role played in cell biology by some of them, like NMN and NR, has emerged [4], [31]. In contrast, despite a deep genetic and biochemical knowledge on NAD biosynthetic enzymes, only a partial overview exists on their activity distribution among mammalian tissues. This is essentially due to the lack of suitable assays to measure their activity in crude extracts. Most current information arises indeed from protein or mRNA quantification experiments. On the other hand, an overview on the NAD biosynthesis physiology in mammalian organisms can be gathered from classic studies [32]–[34], indicating that the amidated and deamidated routes display different relative proportions depending on the tissue. In liver, for example, all enzymes of the pathway illustrated in Figure 1 are known to be present, allowing conversion to NAD of all NAD precursors from nutritional sources, possibly to re-fuel the whole organism with NAD precursors through the bloodstream [33], [34]. In other tissues, conversely, different enzyme levels are likely to reflect particular and intrinsic metabolic needs, also depending on the availability of exogenous pyridine source(s). Tryptophan is a recognized source for *de novo* NAD synthesis (Figure 1), but generally considered insufficient to sustain normal NAD homeostasis [25]. Most NAD in mammals is synthesized from Nam via the amidated route. Liver again, with its elevated NAD turnover, represents a crucial tissue where Nam recycling prevails and NAD re-synthesis is regulated by nicotinamide phosphoribosyltransferase (NamPRT) [35], also based upon a circadian transcriptional control by the clock machinery [5].

In this work we focused on direct measurements in tissue extracts of the activity levels of the three NMNAT isozymes [18] and NADS, using the C57BL/6 mouse as a model organism in view of its wide use for the study of human pathologies. In parallel, we devised suitable assays to measure the tissue levels of these enzymes' reactants, including NMN, NaMN, and NaAD. We combined these data to achieve a rational metabolic reconstruction in each tissue and, as a result, we could observe remarkable tissue-specific differences. Such a comprehensive dissection of the two alternative NAD biosynthetic routes operating *in vivo* within a mammalian organism appears equally important both from the basic point of view and for the rational treatment of NAD-related diseases, *e.g.* by compensating the side-effects of pharmacological systemic inhibition of NAD biosynthesis, via co-supplementation of appropriate NAD precursors that can boost the biosynthetic capacity of alternative routes for NAD synthesis available in selected tissues [12], [36].

## Materials and Methods

### Tissue collection

The animal work was carried out in accordance with the UK Animals (Scientific Procedures) Act of 1986 (under project licence: PPL 40/3482) and approved by the University of Nottingham Ethical Review Committee. Wild-type C57BL/6 mice of age 5–8 months were maintained at the University of Nottingham and humanely killed by cervical dislocation. The tissues were rapidly collected and snap-frozen in liquid nitrogen. Total blood was collected in heparin tubes and frozen likewise. These samples were then ground in liquid nitrogen, weighed in aliquots, and stored at −80°C. All data in this work are referred to grams N_2_-powder ( =  fresh weight tissue).

### Extraction protocols

Tissue N_2_-powders (20-200 mg aliquots) were added with 6 ml/g of 1.2 N HClO_4_ containing 50 µM cAMP (300 nmol/g) as internal standard, and thawed by vortexing. The perchloric extracts were sonicated (3×15 sec at 50 watts with 0.5-sec pulses), and centrifuged (16,000×*g*, 10 min at 4°C). The supernatants were neutralized by adding 0.29 vol of 3 M K_2_HPO_4_ and centrifuged again to remove the precipitated KClO_4_. The clear supernatant referred as neutralized extract was used for subsequent analyses of metabolites (see below). Alternatively, for enzyme assays, the tissue N_2_-powders (50-200 mg) were added with 10 ml/g of 50 mM HEPES/KOH buffer, pH 7.5, containing 20 mM NaF, 1 mM DTT and PMSF, 0.02 mg/ml each leupeptine, antipain, aprotinin, chymostatin, pepstatin. Blood metabolites were extracted using 2 ml/g buffer. After thawing on ice, the crude homogenates were sonicated as above, and immediately used (see below). Before NMNAT assays, a Chelex-treatment was carried out [18]. Protein contents were quantified with the Bio-Rad Protein Assay; their average amounts were (mg/g tissue ± standard error, n≧4): 85±14 (small int.), 64±6 (skel. muscle), 86±11 (spleen), 102±13 (heart), 70±11 (lung), 103±9 (kidney), 76±6 (brain), 135±15 (liver), and 142±6 (blood).

### Pyridine dinucleotides and adenine nucleotides

The endogenous nucleotides NAD, NaAD, ATP, AMP, and the internal standard cAMP, were quantified by UV C18-HPLC analysis both under reverse-phase and ion-pairing conditions, after injection of 20 to 100 µl aliquots of each neutralized extract. The reverse-phase separation was as described [37]. The ion-pair chromatography was as follows. A Supelcosil LC-18-S column (5 µm; 250×4.6 mm) was equilibrated with 100 mM potassium phosphate, pH 6.0, containing 8 mM tetrabutylammonium hydrogen sulfate (buffer A) and eluted at flow-rate of 1 ml/min under the following gradient conditions: 4 min at 100% A; 12.5 min up to 15% B (buffer A plus 30% methanol); 23.5 min up to 90% B; 7 min hold at 90% B, followed by re-equilibration in buffer A. The column temperature was 8°C and the eluate absorbance was monitored by a diode-array detector (Shimadzu SPD-M10A). The two methods allowed distinctive separation of the peaks of interest that were identified by UV spectral analysis and coelution with appropriate standards, in the following elution order: NAD, AMP, NaAD, ATP, cAMP for the ion-pair separation; ATP, AMP, NaAD, NAD, cAMP for the reverse-phase separation. For quantitation, the integrated peak areas, expressed by the Shimadzu LCsolution v1.24 software used as ‘µAU*sec’, were first converted into ‘mAU*ml’ by appropriate factoring, then into nanomoles dividing by the appropriate extinction coefficient at 260 nm (18.6 mM^−1^ cm^−1^ for NAD and NaAD; 15.4 mM^−1^ cm^−1^ for ATP, AMP, and cAMP). The extraction yield calculated from cAMP recovery was ≧95% in all samples.

### Pyridine mononucleotides

**Figure 2:**
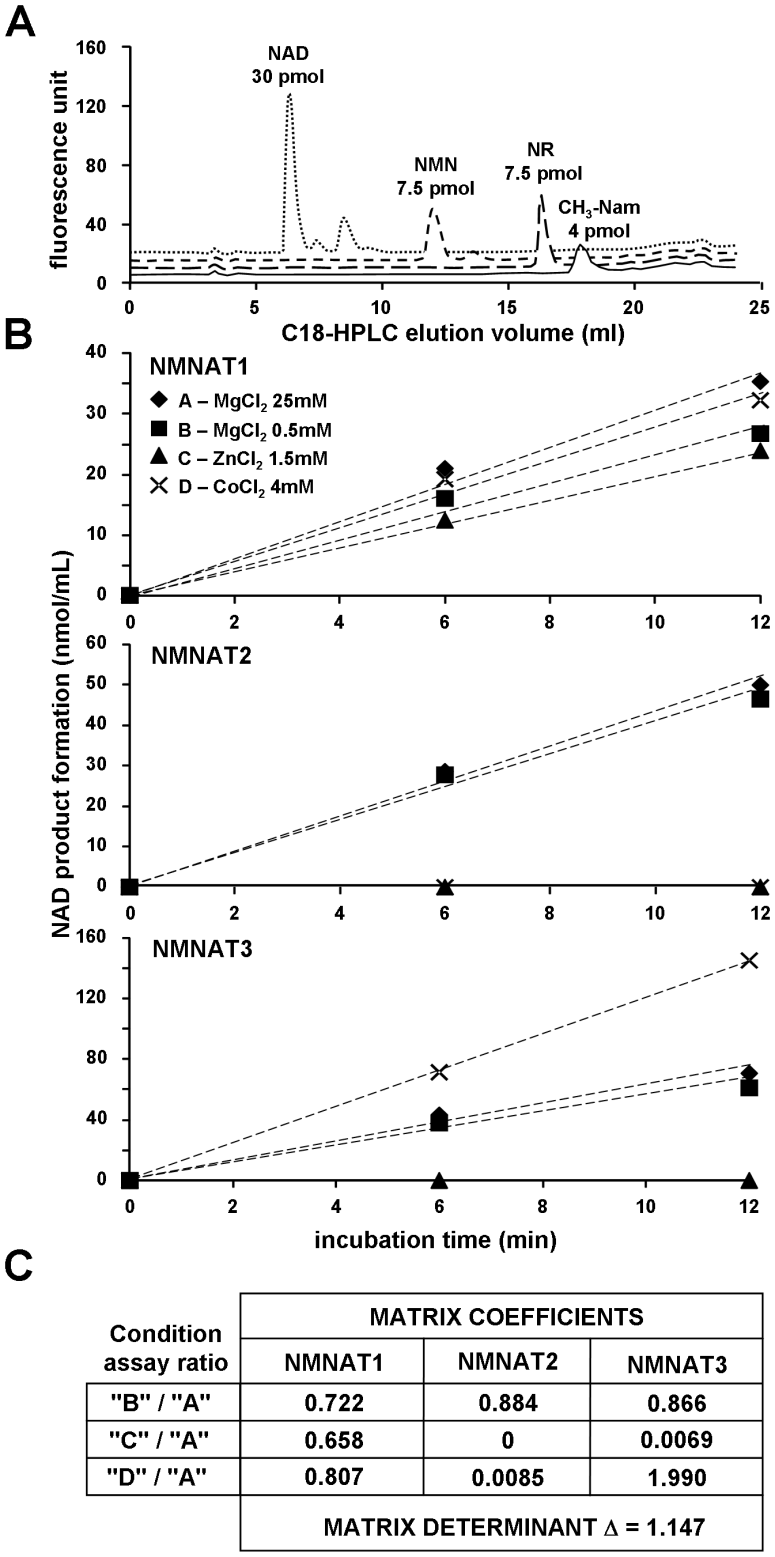
Spectrofluorometric analysis of pyridine metabolites, and discrimination assay of NMNAT isozymes. A, C18-HPLC fluorescence profiles (excitation 380 nm, emission 440 nm) of pure standard pyridine metabolites after chemical derivatization with acetophenone. By this procedure, NMN, NAD, NR, and CH_3_-Nam are all converted into the respective highly fluorescent derivatives. B, discrimination assays of mouse NMNAT isozymes; C, calculation matrix. Each isozyme (pure recombinant) was assayed under four conditions exploiting its own metal ion dependence, *i.e.* in the presence of 25 mM MgCl_2_ (♦, condition A  =  arbitrary reference), or 0.5 mM MgCl_2_ (▪, condition B), or 1.5 mM ZnCl_2_ (▴, condition C), or 4 mM CoCl_2_ (×, condition D). The NAD product formation was evaluated by a NAD cycling assay (see Methods). The reaction rates shown were needed to generate the matrix coefficients used in this work to calculate the individual activities of the three mouse NMNAT isozymes in the tissue extracts. The matrix determinant in this example is 1.147 (absolute value) [18].

Both NMN and NaMN were quantified by spectrofluorometric C18-HPLC analysis after treating each neutralized extract as follows. Typically, four mixtures were set up, containing 50 mM potassium phosphate buffer, pH 7.5, 1 mM MgCl_2_, 2.5 mM ATP, 4 mM NH_4_Cl, and 2 to 20 µl aliquots of neutralized extract. One mixture (Mix 1) was used as such, while the other three mixtures were added with either a known amount of NMN spike (Mix 2), or 10 µg *Francisella tularensis* NMN synthetase (*Ft*NadE, EC 6.3.1.-, pure recombinant [38], 1.5 nmol/min) (Mix 3), or 10 µg *Ft*NadE and a known amount of NaMN spike (Mix 4). The final volume was 50 µl and the two spikes ranged from 5 to 20 pmol. The four replicates were incubated at 37°C for 10 min, boiled at 100°C for 3 min, and derivatized with acetophenone as described [30], leading to a final 250-µl volume at the end of the procedure. Aliquots of 195-µl were injected onto HPLC either immediately or after storage at 4°C, since the fluorescent derivatives obtained under these conditions are chemically stable for days. A Shimadzu LC-10AVP HPLC system holding a Teknokroma Tracer Extrasil ODS2 column (5 µm; 250×4.0 mm) was connected to a Perkin Elmer LS-45 spectrofluorometer set at excitation and emission wavelengths of 380 and 440 nm, respectively. Column equilibration was performed using 100 mM potassium phosphate buffer, pH 2.1, containing 10% acetonitrile (buffer C). The elution gradient was: 6 min at 100% C, 5 min up to 100% D (buffer C plus 40% acetonitrile), 6 min hold at 100% D, followed by re-equilibration in buffer C. The column temperature and the flow-rate were 25°C and 1.5 ml/min, respectively. For quantitative calculation, the integrated peak areas corresponding to NMN arising from Mix 1 and Mix 2 were compared based on the known amount of NMN added. Mix 1 and Mix 3 were compared for NaMN quantitation, and calculation was based on their difference. Mix 4 was the experimental control of *Ft*NadE activity. This procedure allowed highly sensitive detection of NMN directly, and of NaMN indirectly, *i.e.* after quantitative conversion into NMN (detection limit of ∼0.05 pmol). The derivatization chemistry allows direct analysis also of NR, N-methyl-Nam, and NAD (Figure 2A). Finally, being *Ft*NadE active on both NaMN and NaAD substrates [38], indirect analysis of NaAD in parallel to NaMN can be performed by using the same procedure without modification.

### Glutamine and glutamate

The tissue levels of both glutamine and glutamate were determined by the SIGMA GLN-1 kit, using 10–50 µl aliquots of each neutralized extract above.

### Phosphate and polyphosphate-compounds

The method consisted on (i) anion-exchange HPLC separation, (ii) post-column derivatization of free Pi, or of bound Pi released after cleavage of phosphoanhydride bonds, (iii) photometric detection at 780 nm of the Pi-molybdenum adducts formed [39]. A Beckman GOLD HPLC system holding a Tosoh Bioscience TSKgel DEAE-2SW column (5 µm; 250×4.6 mm) was connected post-column to a reagent pump and a reaction oven. The column was thermostatted at 20°C and equilibrated at 1 ml/min with 1 g/l EDTA-Na_2_, pH 4.6, containing 75 mM KCl (buffer E). Elution was carried out by a 20-min linear gradient up to 100% buffer F (1 g/l EDTA-Na_2_, pH 4.6, 300 mM KCl), followed by 5 min holding at 100% F and re-equilibration in buffer E. The post-column analysis was achieved by mixing the eluate, first with a molybdenum reagent solution [39] through the reagent pump (0.5 ml/min), then by fluxing it through a coiled loop (3 m length, ∼0.5 ml internal vol) inserted in the reaction oven at 170°C, and finally through the HPLC detector for absorbance reading. Prior calibration was carried out by injecting pure standard solutions of Pi, PPi, and ATP, that showed good separation and comparable absorbance after derivatization (calculated ε_780 nm_ of 16–18 mM^−1^ cm^−1^ for all the Pi adducts formed), thus providing easy detection of down to ∼5 pmol each compound above. Subsequent injection of tissue extracts (up to 150 µl each) allowed quantitation of endogenous Pi and ATP. The PPi tissue levels, that could not be detected by this analytical system, were calculated by the following equation:
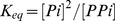
(1)assuming a near-equilibrium condition arising from physiological PPase activity. A *K*_eq_ value of 1350±0,1 M was used [40]; furthermore, the measured Pi tissue levels that were obtained as nmol/g were assumed as micromolar concentrations.

### NMNAT and NADS assays

For NMNAT, the four typical assay mixtures (A–D, Figure 2B) allowing total and isozyme-specific activity discrimination were prepared as reported [18], but in 0.2 ml final volumes, and using as the condition “B” an higher MgCl_2_ concentration (0.5 mM instead of 0.05 mM). This modification was introduced to improve activity detection, after verification that matrix calculation was similarly effective under these new conditions (Figure 2C). NMN was added as the last component to start the reaction. The NADS activity was assayed in 0.2 ml reaction mixtures containing 50 mM potassium phosphate buffer, pH 7.5, 100 mM KCl, 20 mM NaF, 5 mM MgCl_2_, 4 mM ATP, 20 mM L-glutamine, and 1 mM NaAD (reaction start). The assay mixtures for both enzymes contained 15–80 µl homogenate (0.3–5 mg/ml final protein concentration). During incubation at 37°C, reaction aliquots were collected at different times (0, 5, 10 min typically; up to 4 h for blood), and treated with HClO_4_ and K_2_CO_3_ as described [37]. The NAD product was measured by a fluorescence cycling assay [41] carried out on a 96-well BioTek HTTR plate reader. Each well was loaded with appropriate volumes of reaction stops (2 to 20 µl, with or without addition of known amounts of NAD spikes) diluted to 150 µl with water, further added with 100 µl of fresh cycling reagent [41]. The calibration curves obtained by using the internal standard method showed linearity (r^2^>0.99) up to 5 pmol of NAD detected. Therefore, NAD quantitation was always carried out below this limit. Activity rates were calculated from tangent lines in the linear region of plots of product accumulation versus time, and referred as nmol/min/gram of tissue (“maximal rates”). These values were linear *versus* increasing enzyme amounts assayed in the range above reported, and linearity was maintained in homogenates previously centrifuged and desalted by gel filtration.

### Calculation of enzymes' physiological rates and NAD half-life

The *in vivo* estimates of NMNAT and NADS activity in the NAD synthesis direction were obtained by data fitting into the steady-state kinetic equations (2) and (3) (modified, respectively, from IX-89, p. 564, and IX-279, p. 715, in ref [42]). Equation (2) was used for NMNAT isozymes. Equation (3) was used for NADS.
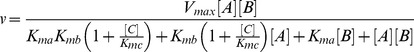
(2)

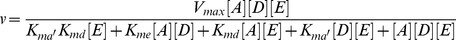
(3)where [A], [B], [C], [D], and [E] are the concentrations of ATP, NMN, NaMN, NaAD, and Gln, respectively, assuming the tissue values “nmol/g” as equal to micromolar; *V*_max_ values are approximated as the “maximal rates” above; *K*_ma_, *K*_mb_, *K*_mc_ are the Michaelis constants of NMNAT isozymes, respectively, for ATP (33.5 µM NMNAT1, 82 µM NMNAT2, 39 µM NMNAT3), for NMN (25.2 µM NMNAT1, 38.5 µM NMNAT2, 117.6 µM NMNAT3), and for NaMN (67.7 µM NMNAT1, 14.5 µM NMNAT2, 111 µM NMNAT3) [18], [19]; *K*_ma'_ *K*_md_, *K*_me_ are the Michaelis constants of NADS, respectively, for ATP (154 µM), NaAD (108 µM), and Gln (2170 µM) [43]. Note that [B] = NMN and [C] = NaMN, and their Michaelis constants, were inverted when using equation (2) for the evaluation of the deamidated reaction catalyzed by NMNAT.

The NAD half-life values were calculated by the following equation:
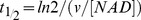
(4)where *v* is the estimated physiological rate of total NAD synthesis, assumed to be equal to the rate of NAD degradation under steady-state conditions, and [NAD] is the measured NAD steady-state concentration in each tissue.

## Results

### NMNAT and NADS activity profiling in mouse tissues

**Figure 3:**
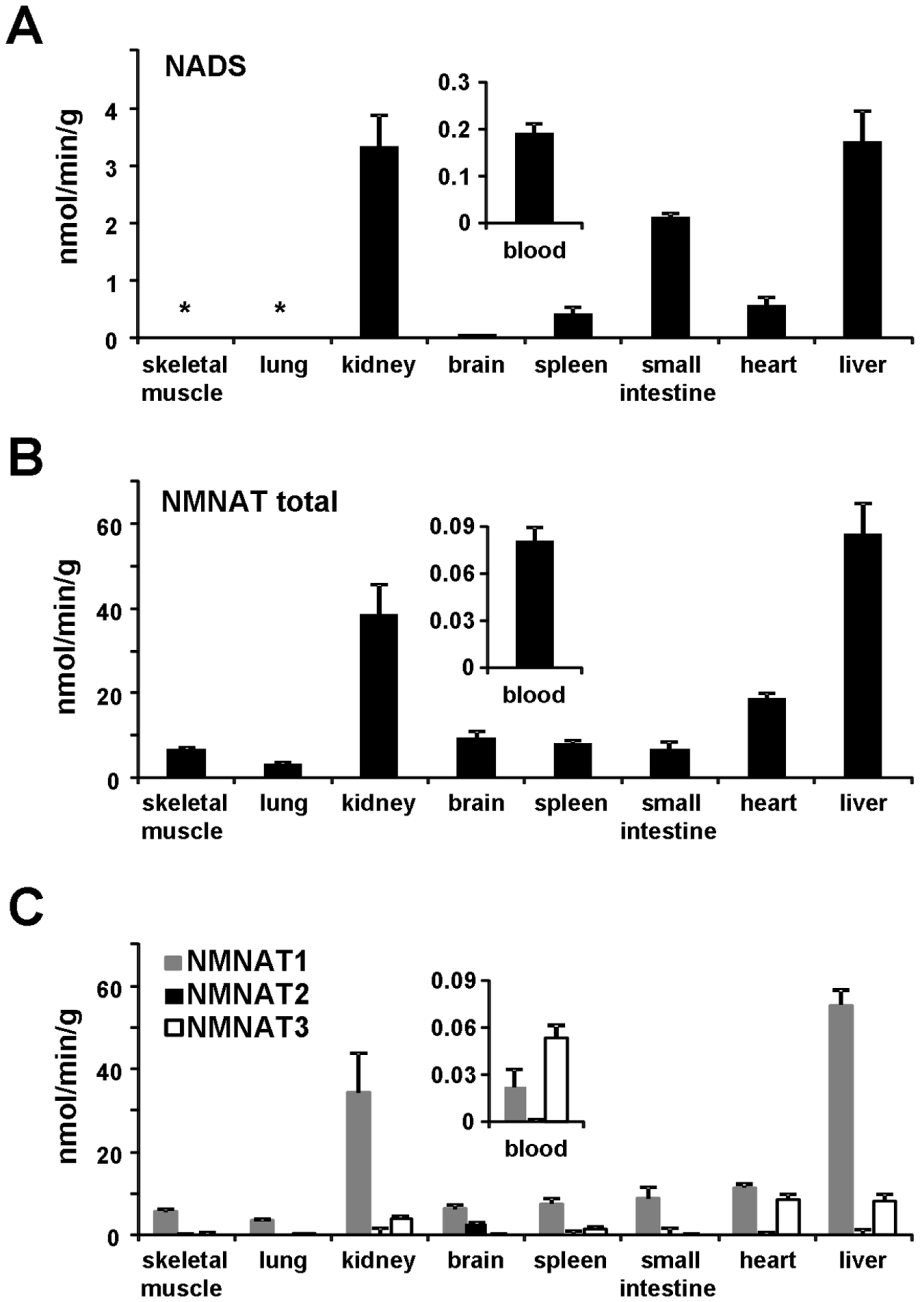
*In vitro* activity profiling of NMNAT and NADS in mouse tissues. A, NADS activity; B, total NMNAT activity; C, isozyme-specific NMNAT activity. Insets show in an amplified scale the activity profile of whole blood. Rates were measured at a saturating concentration of all substrates, and represent the mean ± standard error from at least duplicate analysis. (*) not detectable (*i.e.*, ≤0.005).

We first measured the total NMNAT and NADS activities in several tissue homogenates from the C57BL/6 mouse (Figure 3A, B). Their relative levels varied between tissues, and the highest activity values for both enzymes were observed in liver and kidney. Moreover, NMNAT activity was ubiquitously distributed in contrast to NADS, whose activity levels were barely detectable in brain, and below the detection limit both in lung and skeletal muscle, suggesting a limited NAD synthesis capability through the deamidated route in these tissues. The observed tissue distribution of NADS activity parallels its mRNA expression, which was reported to be high in small intestine, liver, and kidney, and remarkably lower or absent in other tissues [44]. Figure 3 also highlights significantly higher values of total NMNAT activity in most tissues as compared to NADS. Two exceptions were represented by small intestine, where NADS was rather similar to NMNAT, in keeping with the selective, high-level mRNA expression in this tissue [44], and by blood, where NMNAT activity showed indeed the lowest absolute value, even lower than local NADS (Figure 3A, B insets). The levels of the two enzyme activities measured in blood are in good agreement with those reported in erythrocytes [45], [46], and their low values relative to the rest of examined tissues, indicate negligible interference by blood contamination on the non-perfused tissue samples.

Finally, we performed the discrimination assays [18] on the same tissue extracts, in order to achieve activity determination of individual NMNAT isozymes. As depicted in Figure 3C, the three isozymes showed differential contributions to total NAD formation, being never simultaneously present at comparable levels in individual tissues. Nuclear NMNAT1 activity was ubiquitous and predominant in all examined tissues, with the exception of blood (Figure 3C inset). Cytosolic NMNAT2 activity was clearly detectable only in brain. The mitochondrial NMNAT3 isozyme was predominant in blood, as expected from its reported high level in isolated erythrocytes [46], and also present, along with NMNAT1, in heart, liver, kidney, and spleen. Also this activity profile appears consistent with the reported mRNA profiles [16], [47]–[50].

### Metabolites analysis and tissue profiling

**Figure 4:**
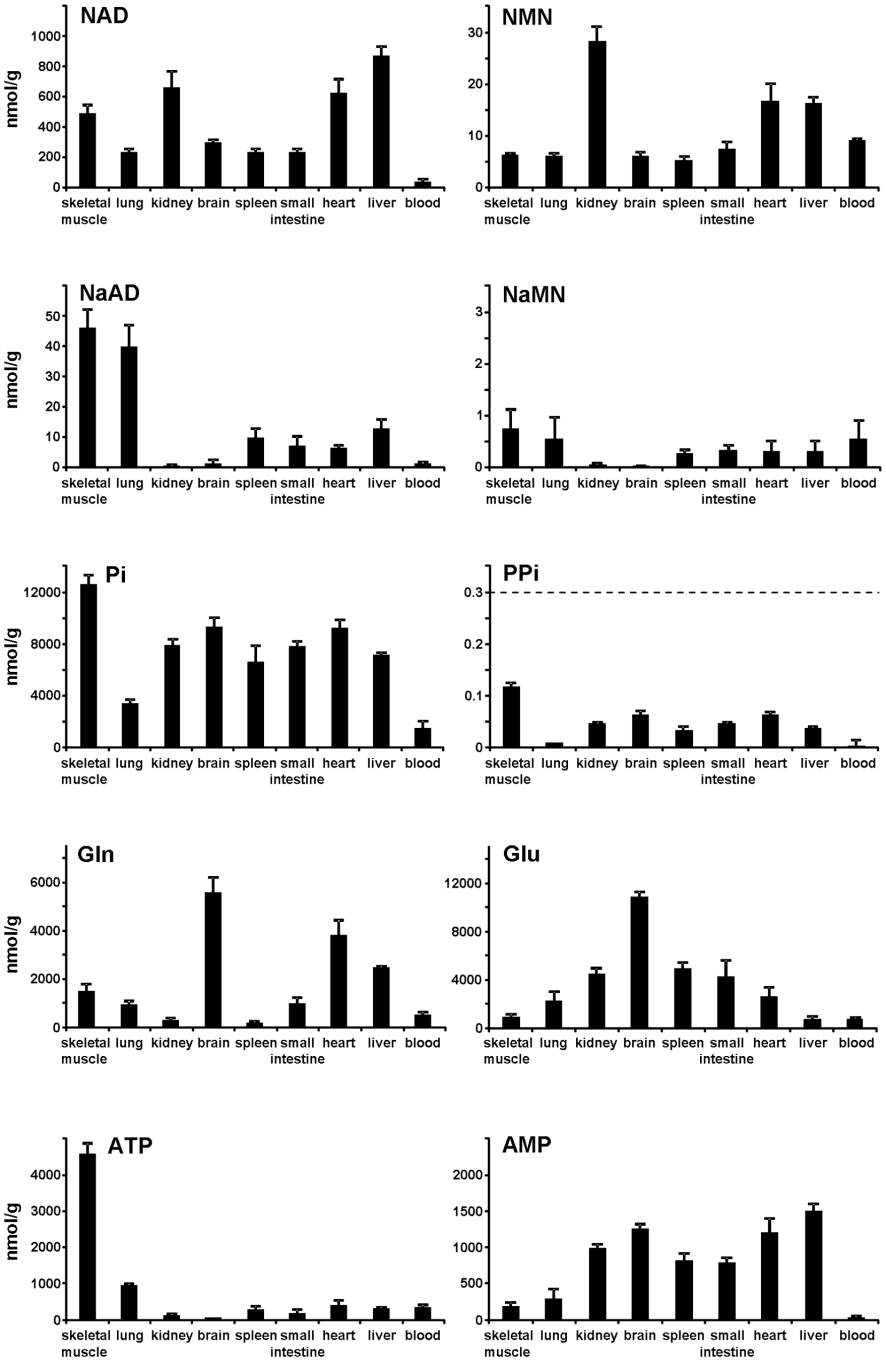
Overall profiling of the reactants levels of the NADS- and NMNAT-catalyzed reactions. Values arise from multiple analyses (mean ± standard error, n≧2) and are indicated as nmol per gram tissue. Dotted line, detection assay limit; the indicated PPi values are calculated from the corresponding Pi contents, as described in Methods, assuming rapid enzymatic equilibration by inorganic PPase.

As detailed in Methods, in order to analyze the metabolites of interest, we used mouse tissues immediately frozen *post mortem* and processed without prior thawing. The resulting extracts were first directly analyzed by UV C18-HPLC. This method, however, was inadequate for NMN and NaMN, due to their low physiological levels and low specific UV absorption, as well as poor chromatographic separation. We thus determined NMN by a fluorometric method following derivatization of its alkylpyridinium group with acetophenone [30]. We used the same method to detect NaMN, after its stoichiometric conversion to NMN by means of a bacterial recombinant NMN synthetase [38]. We developed a novel C18-HPLC separation for quantitation of the mononucleotide fluorescent derivative (Figure 2A). NaMN was detected as NMN, by subtracting the endogenous NMN background. The procedure resulted feasible and comparably sensitive with respect to conventional LC/MS methods, with a detection limit as low as ∼0.05 pmol of NMN under the conditions described. Finally, we investigated on the remaining components of the two reactions catalyzed by NMNAT and NADS, *i.e.* PPi and the couple glutamate/glutamine. As to the former compound, we performed an anion-exchange HPLC analysis with post-column derivatization [39]. Despite the sensitivity of this method, the PPi levels were undetectable, *i.e.* below 0.3 nmol/g, whereas endogenous Pi was readily measured in all tissues (Figure 4). These results are in keeping with the physiological *K*_eq_ value of the inorganic PPase reaction [40], therefore we resorted to estimate PPi from the measured Pi tissue levels (see Methods).

Figure 4 summarizes the measured steady-state levels of the various substrates and products of both NMNAT and NADS reactions in mouse tissues. The NAD levels generally ranged between 200 and 900 nmol/g. Only blood showed a lower value of ∼40 nmol/g corresponding to nmol of NAD per ml of blood. The highest NAD levels were observed in liver, kidney, heart, and skeletal muscle, in keeping with reported values [51]. The NMN levels ranged from 5 to 30 nmol/g, with highest amounts in liver, kidney, and heart. Of note, both NAD and NMN levels in blood appear in good agreement with those determined in isolated mouse erythrocytes [29]. The measured tissue levels of NaAD and NaMN were at least one order of magnitude lower than NAD and NMN, respectively. They were relatively low in kidney and brain, and particularly high in lung and skeletal muscle (Figure 4). The PPi levels ranged from 0.01 to 0.1 nmol/g. The glutamate and glutamine levels ranged from 700 to 11,000 nmol/g, with the exception of lower glutamine levels in kidney, spleen, and blood (200–500 nmol/g). Finally, Figure 4 bottom, shows the tissue profiles of ATP and AMP. The total adenylate pool analysis (including ADP, not shown) yielded highest values in skeletal muscle, as expected, and lowest in blood. In all tissues except lung, blood, and skeletal muscle, a relatively low ATP level and high AMP could be observed.

### Translating *in vitro* results into *in vivo* metabolic predictions

In principle, for *in vivo* evaluation of individual enzymatic steps within a known metabolic pathway, two parameters need to be established, *i.e.* (i) how far the physiological steady-state levels of substrates and products are kept from the chemical equilibrium, and (ii) the rate of the reaction under steady-state conditions (physiological rate). In this study, both parameters can be derived by combining the data reported above and the available kinetic properties of the two enzymes. Indeed, NMNAT catalyzes a thermodynamically reversible reaction, with an equilibrium constant close to unit [16], [52], and all three mouse isozymes show similar Michaelis-Menten constants for their substrates, in the sub-millimolar range [18], [19]. In contrast, the NADS-catalyzed reaction is considered physiologically irreversible [14], [53]. The equilibrium constant and substrate affinities of mouse NADS are not available, but the first parameter can be approximated to exceed 10^6^ based on reported standard free energy change [54], whereas affinities can be assumed to be similar to those of human NADS [43].

**Figure 5:**
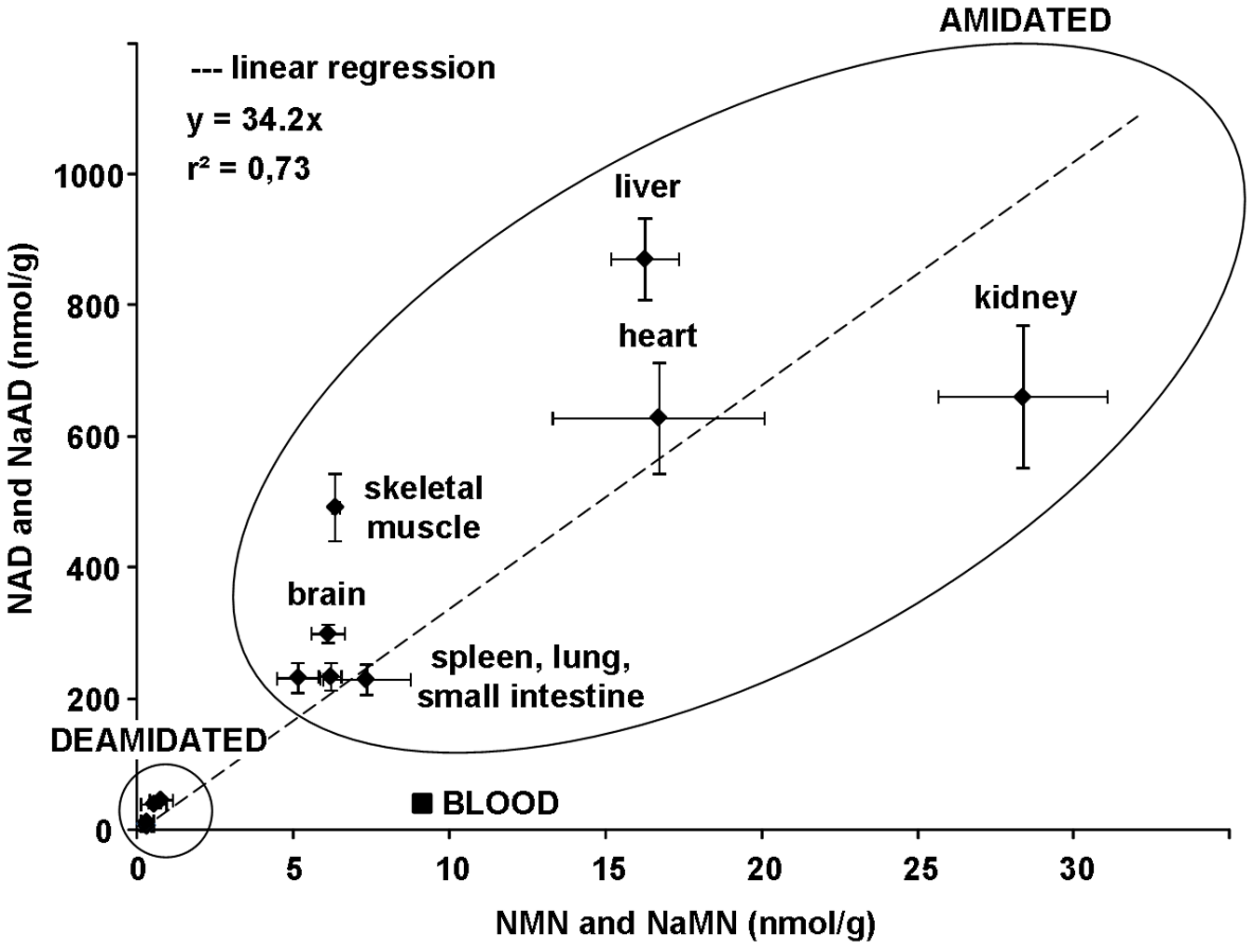
Correlation between the physiological levels of products (NAD or NaAD) and substrates (NMN or NaMN) of the NMNAT-catalyzed reaction in different mouse tissues. Average values ± standard errors are those depicted in Figure 4. The “AMIDATED” refers to grouped NAD and NMN levels in the indicated tissues; the group “DEAMIDATED” refers to levels of NaAD and NaMN (smaller circle) in the same tissues (not detailed). The linear relationship in different tissues between the steady-state levels of NAD and NaAD relative to NMN and NaMN, respectively, is highlighted. The “outlier” square symbol (▪) represents blood, where diverging values were observed compared to all other examined tissues.

From data in Figure 4, we first derived the physiological product/substrate ratios and reaction quotients (Table 1). As for the NMNAT-catalyzed reaction, a noticeable regularity emerged among tissues. In fact, the levels of the dinucleotide product (NAD or NaAD), in spite of fairly scattered and uneven values observed in different tissues, appear constantly larger than the mononucleotide substrates: tissues with higher NAD (or NaAD) levels show NMN (or NaMN) levels that are proportionally higher. Therefore, in all tissues, the [NAD]/[NMN] ratio appears remarkably similar to the [NaAD]/[NaMN] ratio (Table 1), consistent with the notion that the same enzyme, NMNAT, catalyzes both the amidated and deamidated reactions with comparable efficiency. Moreover, the dinucleotide/mononucleotide ratio values (Table 1) notably showed a similar magnitude of ∼35 in most tissues (Figure 5), suggesting that NMNAT equally modulates the reaction in different districts of the mammalian organism. The only exception was represented by blood, where much lower values were observed for both ratios (Figure 5), in keeping with the unique tendency for NMN and NaMN to accumulate, in this tissue, relative to NAD and NaAD. The NMNAT reaction quotients, estimated by taking into account the physiological concentrations of the two other reaction components, ATP and PPi, resulted to be invariably similar among tissues, with values about two orders of magnitude below the known equilibrium constant. Only in blood, a much lower value was calculated, about four orders of magnitude below the known equilibrium constant (Table 1). Regarding the NADS reaction, conversely, a different scenario arose, and the ratio values showed in general a rather higher heterogeneity, still with some regularity. In particular, three distinct situations were evidenced regarding the tissue NADS reaction quotients: (i) very low values (lung, skeletal muscle, blood), (ii) very high values (brain and kidney), and (iii) intermediate values (remaining tissues) (Table 1).

**Table 1:** Product/substrate ratios and physiological reaction quotients in mouse tissues.

	NMNAT reaction (*K*_eq_∼1)				NADS reaction (*K*_eq_>10^6^)			
	[NAD]/[NMN]	[NaAD]/[NaMN]	[PPi]/[ATP]	Physiological reaction quotient^a^	[NAD]/[NaAD]	[AMP][PPi]/[ATP]	[Glu]/[Gln]	Physiological reaction quotient
skeletal muscle	78	62	0.03•10^−3^	0.002	11	0.005	0.6	0.03
lung	38	74	0.01•10^−3^	0.001	5,9	0.003	2.5	0.04
kidney	23	16^b^	0.39•10^−3^	0.009	∼1000	0.39	15	∼5800
brain	49	100^b^	1.29•10^−3^	0.063	∼1000	1.64	2.0	∼3200
spleen	45	36	0.12•10^−3^	0.005	24	0.10	26	60
small intestine	31	22	0.23•10^−3^	0.007	32	0.18	4.4	26
heart	38	20	0.16•10^−3^	0.006	99	0.19	0.7	13
liver	54	42	0.12•10^−3^	0.006	68	0.18	0.3	4
blood	4	2^b^	0.01•10^−3^	0.00002	∼50	0.0002	1.5	∼0.01

a Values are calculated for the “amidated” reaction catalyzed by NMNAT; the corresponding values for the “deamidated” reaction are of the same magnitude.

b NaMN and/or NaAD determined values are very close to the assay detection limit.

By evaluating altogether these results, based on the relative distance from the equilibrium constants, it appears that NMNAT limits the rate of NAD synthesis in blood but not in the other tissues examined, while NADS is limiting to different extents depending on the tissue type. On the other hand, all calculated tissue reaction quotients of NMNAT and NADS catalyzed reactions are clearly below the respective chemical equilibrium values, indicating that *in vivo* these enzymes are bound to catalyze NAD synthesis at higher rate than the reverse reactions. Accordingly, in all examined tissues, the PPi levels were too low to support the reverse reactions at significant rate (Figure 4). Thus, on this basis, subsequent estimates of the enzymes' intracellular rates of product accumulation were focused on the forward reactions only. Such rate estimations were carried out on NADS (deamidated route), as well as on individual NMNAT isozymes (amidated and deamidated routes). The measured tissue levels of all substrates of each reaction (Figure 4) were fitted into the steady-state kinetic equations (2) and (3) as described in Methods. This allowed conversion of the activity values measured *in vitro* under conditions of “maximal rate” (Figure 3), into the tissue *in vivo* “physiological rates” depicted in Table 2 and Table 3. Although all substrates were included in the calculation, we noted that mainly the pyridinic substrates (NMN or NaMN for NMNAT, and NaAD for NADS) influenced significantly the resulting rate value, being the other substrates almost saturating. Furthermore, since NMN and NaMN are simultaneously present *in vivo* along with the NMNAT enzyme of which both are substrates, the rate equation (2) was appropriately corrected to take into account the co-substrate inhibition effect. As outlined in the Discussion section, these *in vivo* estimates assume the absence of any enzyme regulation other than the substrate concentration.

**Table 2:** Amidated route: estimates of NMNAT isozymes' physiological activity.

	NMNAT1^a^	NMNAT2^a^	NMNAT3^a^	Total NMNAT
	nmol/min/g	nmol/min/g	nmol/min/g	nmol/min/g
skeletal muscle	1.12	nd	nd	∼1.1
lung	0.64	nd	nd	∼0.6
kidney	14.1	nd	0.57	∼15
brain	0.73	0.14	nd	∼0.9
spleen	1.14	nd	0.051	∼1.2
small intestine	1.70	nd	nd	∼1.7
heart	4.17	nd	0.97	∼5.1
liver	19.5	nd	0.90	∼20
blood	0.0054	nd	0.0035	∼0.009

a Values are calculated from the steady-state kinetic equation (2) described in Methods, using the NMNAT isozyme activities measured at saturating substrates (Figure 3C) as *V*_max_ values, the NMN and ATP levels (nmol/g, Figure 4) as micromolar concentrations, and the *K*_m_ values from [18]; nd, not detectable.

**Table 3:** Deamidated route: estimates of NMNAT and NADS physiological activities.

	NMNAT total rate^a^	NADS rate^b^	Combined values
	nmol/min/g	nmol/min/g	nmol/min/g
skeletal muscle	∼0.05	≤0.001	≤0.001
lung	∼0.02	≤0.001	≤0.001
kidney	∼0.007	∼0.007	∼0.007
brain	∼0.001	∼0.0006	∼0.0008
spleen	∼0.02	∼0.01	∼0.02
small intestine	∼0.03	∼0.07	∼0.05
heart	∼0.05	∼0.02	∼0.04
liver	∼0.17	∼0.23	∼0.20
blood	∼0.0005	∼0.001	∼0.001

a Sum of the three NMNAT isozyme activities individually calculated as in Table 2 but versus the NaMN substrate (*K*_m_ values used are reported in ref [19]).

b Values are calculated from the steady-state kinetic equation (3) shown in Methods, using the NADS activities measured at saturating substrates (Figure 3A) as *V*_max_ values, the NaAD, ATP, and glutamine levels (nmol/g, Figure 4) as micromolar concentrations, and the *K*_m_ values from [43].

The *in vivo* activity profile of individual NMNAT isozymes operating in the amidated route (Table 2), clearly evidenced NMNAT1 as predominant in all tissues, even in blood where *in vitro* “maximal rate” was instead higher for NMNAT3 (Figure 3C inset). Furthermore, the deamidated route (Table 3) showed calculated *in vivo* activities of NMNAT (total isozymes' activity) and NADS substantially matching to each other in all tissues, with the exception of skeletal muscle and lung, where NADS activity is extremely low, as expected from its undetectable *in vitro* “maximal rate” in these tissues (Figure 3A). In general, a comparison of rates in Tables 2 and 3 highlights that, in all tissues, the amidated route is remarkably faster and more uniformly distributed, compared to the deamidated route. The absolute and the percent contribution of either route to the overall rate of NAD biosynthesis are summarized in Table 4.

**Table 4:** Estimated rates of total NAD synthesis (“amidated” plus “deamidated”) in mouse tissues.

	*In vivo* total NAD synthesis rate^a^	amidated route	deamidated route	NAD turnover half-life^b^
	nmol/min/g	relative	relative %	min
skeletal muscle	∼1.1	≧99.9	≤0.1	∼300
lung	∼0.6	≧99.9	≤0.1	∼250
kidney	∼15	99.9	0.1	∼30
brain	∼0.9	99.9	0.1	∼240
spleen	∼1.2	98.4	1.6	∼130
small intestine	∼1.7	97.2	2.8	∼90
heart	∼5.2	99.3	0.7	∼85
liver	∼21	99.1	0.9	∼30
blood	∼0.01	90.9	9.1	∼2850

a Sum of estimated rates of “amidated” (Table 2) and deamidated routes (Table 3).

b Values are calculated by the equation (4) shown in Methods, using the *in vivo* total NAD synthesis rate as “*v*” value (assumed to equal the *in vivo* total NAD degradation rate), and the NAD level in each tissue (Figure 4).

Remarkably, from these data, we could also estimate the physiological NAD turnover rate. We reasoned that, in each tissue, in order to maintain the measured steady-state level of NAD (Figure 4), the total rate physiologically driving NAD biosynthesis should match the overall NAD degradation rate. Hence, the half-life of NAD turnover in each tissue could be calculated, and the results are shown in Table 4. The lowest half-life values were found in liver and kidney (∼30 min), while the highest value was found in blood (∼48 hours). Our estimates are in agreement with the half-life of ∼45 min previously measured in the human cell line D98/AH-2 by using a radioactive double-tracer approach [55]. As compared to radioactive methods, that are subject to a number of drawbacks and limitations, this is a novel approach of wide applicability, that enables a systematic evaluation of NAD half-life in mammalian tissues under different physiological conditions.

### Metabolic reconstruction of the pyridine mononucleotides-NAD pathway in mouse tissues

**Figure 6:**
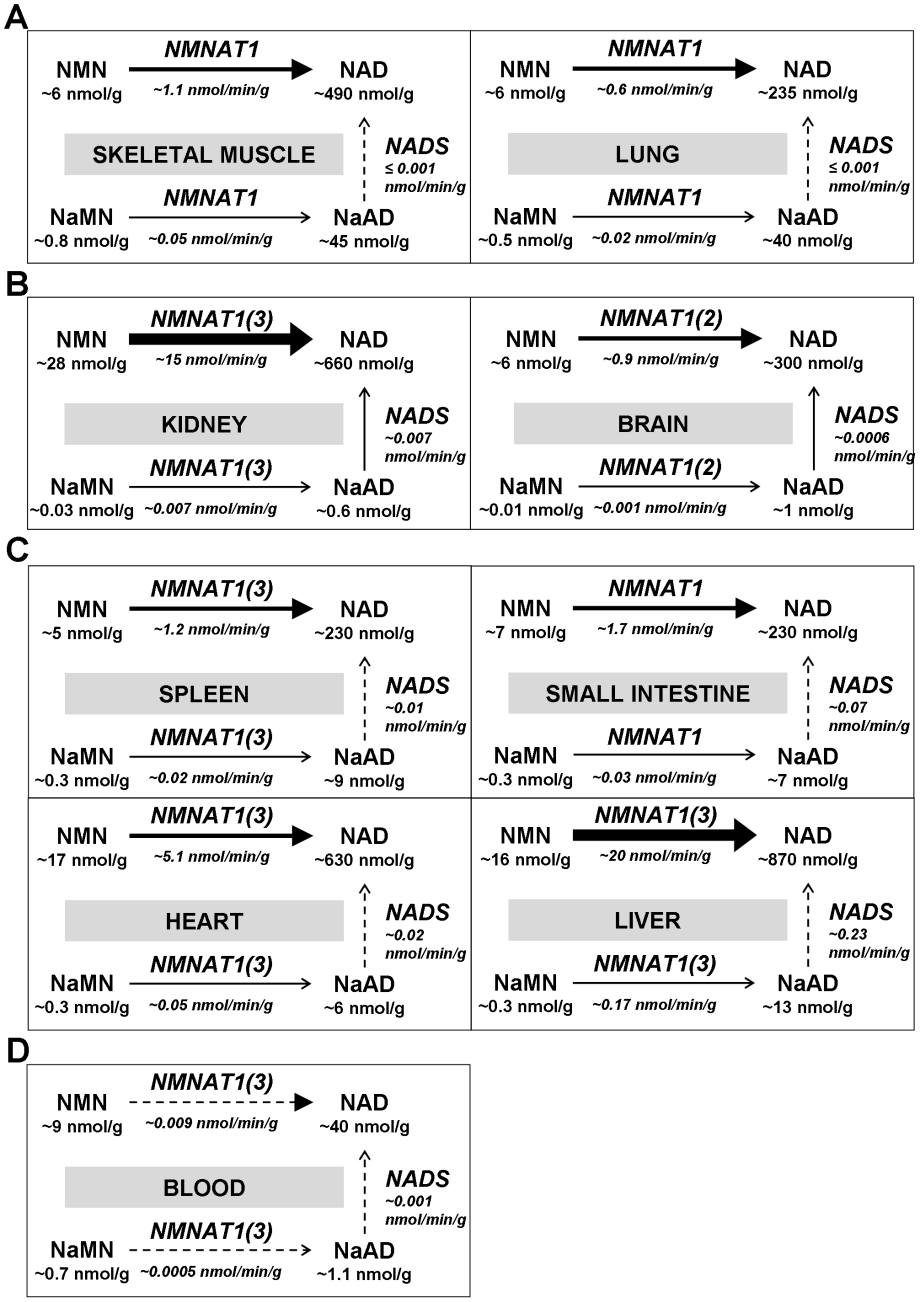
Metabolic reconstruction of the NAD biosynthesis rate in mouse tissues. The nine tissues analyzed are clustered in four distinct groups (panels A, B, C, D). Only the NMNAT and NADS steps of the pathway are shown, together with the steady-state levels of their pyridine reactants (data are taken from Figure 4, Table 2, and Table 3). The *in vivo* rates of NMNAT isozymes were individually estimated, but here only the total rate is indicated, and the predominant and secondary (within brackets) isozyme species is highlighted in each tissue. Dotted arrows indicate the reactions whose physiological reaction quotients fall most prominently below the thermodynamic equilibrium constant (Table 1).

Figure 6 summarizes our tentative metabolic reconstruction based on the above estimates *in vivo*, at the level of the final steps gating NAD synthesis. The nine tissues analyzed were sub-grouped according to their metabolic similarities, as described below.

#### Lung and Skeletal Muscle

In these two tissues (Figure 6A), the amidated route appears largely predominant *in vivo*, showing a similar rate of ∼1 nmol/min/g (Table 2), that nearly represents the total NAD synthesis (Table 4). Regarding the deamidated route, a disproportion between high NMNAT and very low NADS physiological rates is clearly evident (Table 3). A disproportion is also evident by comparing the high physiological reaction quotients of NMNAT with the very low ones of NADS, relative to their equilibrium constants (Table 1). Overall, these data indicate that, in these tissues, NADS activity is limiting for the deamidated route. Interestingly, this is in keeping with the observed accumulation of NaAD and NaMN at relatively high levels (respectively 10% and 17% relative to the local NAD and NMN levels, Figure 4), suggesting the occurrence of possible, yet unknown, physiological roles for the accumulated nucleotides (see Discussion).

#### Brain and Kidney

The estimated physiological rate through the amidated route is ∼15 nmol/min/g in kidney, and ∼1 nmol/min/g in brain (Table 2), representing in both cases ∼99.9% of total NAD synthesis rate (Table 4). Substantially diverging from lung and skeletal muscle, neither step appears rate-limiting in these tissues (Figure 6B). Indeed, the physiological rates of the two steps in the deamidated route appear balanced (Table 3), the physiological reaction quotients appear high for both enzymes relative to the equilibrium constants (Table 1), and both NaAD and NaMN do not appear to accumulate (Figure 4). Overall, the metabolic picture highlights that NAD synthesis in these tissues mainly occurs through the amidated route, and is controlled by the physiological availability of substrates.

#### Spleen, Heart, Small Intestine, and Liver

Also in these tissues, the “amidated” NAD biosynthesis is prevalent (Figure 6C). Indeed, physiological rate estimates through the amidated route of ∼1 nmol/min/g in spleen, ∼2 nmol/min/g in small intestine, ∼5 nmol/min/g in heart, and ∼20 nmol/min/g in liver (Table 2) represent between 97 and 99% of total NAD biosynthesis rate (Table 4). The physiological rates of the two analyzed reactions within the deamidated route are relatively similar in each tissue, and higher than those estimated in all other tissues (Table 3). Accordingly, some tendency for both NaAD and NaMN to accumulate is also evidenced (1–4% of local NAD and NMN levels, respectively, Figure 4). Furthermore, these four tissues showed NADS reaction quotients of intermediate values (Table 1), indicating that NADS is limiting, although less markedly than in lung and skeletal muscle.

#### Blood

In this tissue (Figure 6D), the physiological rate of the amidated route showed the lowest absolute value, amounting to ∼0.009 nmol/min/g (Table 2). In contrast, the estimated rate of the deamidated route appears more similar to other tissues (Table 3). This leads to a relative contribution of the amidated route to the total rate of NAD biosynthesis of “only” 91% (Table 4). Also unique to blood is the very low physiological quotient of the NMNAT reaction, arising from relatively low steady-state levels of pyridine dinucleotides compared to the corresponding mononucleotides (*e.g.*, [NAD]/[NMN] ratio of 4, compared to ∼35 of average tissue, Figure 5). Accordingly, NaAD appears to accumulate in blood like in other tissues, but to a lower extent than NaMN, the latter representing ∼8% relative to the local NMN pool (Figure 4). These data suggest that NMNAT is rate-limiting in blood, although this cannot be taken as conclusive regarding the amidated route, since none of the activities of upstream enzymes has been measured. Conversely, regarding the deamidated route, the rate-limiting NMNAT shows a downstream effect on NADS reaction, that is kept far below its chemical equilibrium in this tissue, similarly to lung and skeletal muscle (Table 1). However, failure to approach the reaction equilibrium in blood cannot be ascribed to a lack of NADS activity (Figure 3A). Notably, the proposed blood metabolic reconstruction, also featuring NMNAT as a limiting enzyme for the deamidated route, is similar to that previously described in human erythrocytes using a different approach [34].

## Discussion

In the redox cell metabolism NAD is used catalytically, *i.e.* it is not degraded while it is continuously cycled between oxidized and reduced forms. Instead, in the NAD signaling usage, NAD is cleaved and its re-synthesis becomes imperative for cell survival. As a result, specific limiting steps in NAD biosynthesis have become targets for rational drug design. Successful drug will block NAD synthesis if no alternative route is available in the target organism or tissue to bypass blockade and counteract the aimed drug action. Thus, rational interception of NAD homeostasis metabolism needs to take into account all NAD synthesizing routes and branches, in order to assess their tissue distribution/partition and to quantitate their relative contribution to total NAD synthesis under variable physiological and pathological conditions.

In this work we addressed such investigation in a model organism, C57BL/6 mouse, under physiological conditions, *i.e.* in the absence of pathology or any treatment. We started from the widespread knowledge that two independent metabolic routes exist in mammals, each ending with an independent enzymatic step. Focusing on these two last steps, we aimed at estimating the rate and steady-state parameters of individual underlying reactions *in vivo*, by using data obtained from *in vitro* measurements of (i) enzymes' activities and (ii) metabolites' levels in different tissues. This “reductionist” approach finally converged into the metabolic reconstruction depicted in Figure 6, that represents the first dissection of the dual pathway for NAD biosynthesis, *i.e.* the “amidated” and “deamidated” routes, physiologically operating at different rates within different districts of the mammalian organism. It is worth noting that our *in vivo* rate estimates of terminal enzymes, NMNAT and NADS, were calculated from the measured steady-state levels of their substrates, NMN and NaAD, that reasonably incorporate the contribution by upstream enzymes in the same branch of the pathway (Figure 1). Thus, as long as the measured substrates' levels are representative of true steady-state conditions, additional investigation on such upstream enzymes does not appear necessary to assess the overall contribution of either route to NAD biosynthesis.

The overall analytical determinations were carried out on appropriately collected mouse tissues, by means of highly sensitive, dedicated assays. The enzymes' maximal rates measured *in vitro* yielded for the first time an extensive synopsis of the tissue levels of the three individual NMNAT isozymes, as well as of NADS. Their distinctive tissue distribution resulted in keeping with previously reported mRNA expression profiles, itself predicting distinctive metabolic features with respect to NAD biosynthesis. Besides, since these *in vitro* rates do not necessarily mirror the *in vivo* rates, in order to calculate the latter, a metabolomic tissue profiling of the physiological steady-state levels of all reactants for such enzyme reactions was assessed in parallel. A new analytical method was devised to unravel the low levels of NAD biosynthesis intermediates like NMN, NaMN and NaAD, yielding for the first time a comprehensive tissue profiling. As to the other reactants, their tissue levels were mostly in keeping with reported data, and also showed consistency between replicates and multiple analyses. Only in some tissues, a partial energy charge depletion was indicated by the observed relative AMP rise and ATP decline (Figure 4). Such a low energy status likely arises from *post mortem* metabolism as a consequence of instant hypoxia [56], [57], despite any attempt to shorten the tissues collection time. On this regard, we could verify by means of appropriate numerical simulations (not shown), that this only marginally affected our results and the proposed metabolic reconstruction.

Furthermore, the reported analytical data have been obtained from tissues partly heterogeneous as to cell-type composition, and after unavoidable disruption of subcellular compartments. This, however, is not different from the approach commonly used in expression studies, and it does not appear to influence significantly the conclusions at the organism level. The rationale of our study is founded on the main assumption that enzymes and reactants are freely accessible to each other in the cell environment. This is justified as far as their cellular distribution is suitably balanced, which appears to be the case for NADS as well as for the differently localized NMNAT isozymes. Indeed, the observed physiological predominance of nuclear NMNAT1, and the NADS cytosolic location, appear both matching with the reported relative abundance of all their substrates in the nucleo-cytoplasm of mammalian cells [36]. Therefore, *in vitro* measurements of average tissue levels of the enzyme reactants and activities, together with a parallel knowledge of all involved enzymes' kinetic parameters, make it possible a realistic assessment of *in vivo* metabolic rates. These latter values in fact, being essentially derived from the steady-state concentrations of enzyme substrates, do not take into consideration any eventual physiological modulation by endogenous small molecule effectors. On this regard, however, it must be noted that the existence of any such “dialyzable” effectors is at present unknown for both NMNATs and NADS, and also that their presence in our tissue extracts could be ruled out by comparing the measured enzyme activity levels in crude and desalted homogenates (see Methods).

The data presented in Table 1 evidenced the tissue [NAD]/[NMN] ratio as a good indicator of the local NMNAT contribution *in vivo*, being its alteration observed only in blood where NMNAT is a limiting enzyme. Likewise, the [NAD]/[NaAD] ratios appear representative of the NADS behavior *in vivo*, in that they parallel the respective physiological reaction quotients. Thus, for both enzymes, the observed pyridinic [product]/[substrate] ratio appears itself, regardless non-pyridine reactants, as a good predictor of the enzyme rate-limiting character. This observation might be of help in several kind of studies, *e.g.* those regarding peripheral nerve diseases, where impaired NMNAT2 enzyme levels are associated with the neurodegeneration process [58].

In all mouse tissues examined in this work, the amidated route resulted the most relevant contributor to overall NAD biosynthesis *in vivo* (Table 4), in keeping with current knowledge for the mammalian organism as a whole. As illustrated in Figure 1, this route can salvage either Nam or NR, or both. While the physiological role of NR in mammals has only recently been addressed [31], Nam is an established NAD precursor, arising both from the diet and from the NAD-cleaving activity of intracellular signalling enzymes, *e.g.* sirtuins, CD38, and PARPs [11]. Widely recognized is also that the latter enzymes are all inhibited by Nam, so that Nam homeostasis *in vivo* requires careful control [7]. This is achieved by suitable recycling of Nam to NAD throughout the sequential action of NamPRT and NMNAT (Figure 1, amidated route), or via Nam methylation and urinary excretion [4], [5], [10], [12]. In view of its inhibitory action, Nam is likely to be a better physiological precursor of NAD than is NR, such that the physiological rates of the amidated route presented in Figure 6 are likely to recapitulate the major contribution by the NamPRT-NMNAT biosynthetic axis. However, a relevant contribution by the other amidated axis, NRK-NMNAT, cannot be discounted in some tissues, like skeletal muscle, where (i) NAD formation from NR is reported [59], [60], and (ii) the alternate deamidated route is almost absent (Figure 6A). On this regard, the results of our spectrofluorometric determinations (see Figure 2A and Methods) allow to exclude any relevant accumulation of NR in all examined tissues (not shown), suggesting an underlying high rate of its removal *in vivo*.

Most importantly regarding the amidated route, the estimated *in vivo* rates were clearly higher in liver and kidney, and clearly lower in blood, compared to the rest of tissues (Table 2). Consistent with the above discussed predominance of the amidated route, liver and kidney exhibited the highest rate of overall NAD turnover, whereas blood showed the lowest value (Table 4). Thus, liver and, somehow unexpectedly, kidney appear both central for the organismal NAD turnover, equally playing as pivotal contributors to amidated NAD recycling. This might be instrumental to divergent functions of these organs in the economy of the mammalian body. In this view, in fact, liver might serve as centre for the conversion of all NAD precursors to Nam, to be further supplied to other organs [12]. In kidney, instead, NAD turnover might be designed to direct Nam, or its catabolic derivatives, to their known urinary excretion [61]. With respect to the organismal NAD homeostasis, blood seems to play a more neutral role, being mainly in charge of transport and delivery of NAD precursors. Taking into account that erythrocytes represent the most abundant blood cell type, the observed lowest rate of NAD turnover in blood can be explained by the lack of nuclei and mitochondria in mature red cells, resulting in a peculiar redistribution of NMNAT isozymes [46], [62] and reduced total NMNAT activity [63].

Regarding the deamidated route for NAD biosynthesis, it showed a marked heterogeneity among tissues. Although in terms of absolute values this route was quantitatively less relevant throughout, as compared to the amidated route, the highest rates were evidenced in liver again, consistent with this tissue's central role for organismal NAD homeostasis, as well as in small intestine, consistent with this tissue's absorption role in the digestion process. Notably, this is the only route for *de novo* NAD synthesis from tryptophan, as well as for the utilization/recycling of dietary Na and Qa precursors (Figure 1). Our data show that usage of such deamidated NAD precursors is relatively negligible within the mammalian organism under physiological conditions. Nonetheless, most examined tissues show an intriguing potential to enhance such usage, if necessary, without the need to resort to over-expression of NMNATs and/or NADS enzymes. Exceptions are lung and skeletal muscle, owing to the observed lack of NADS. Indeed, by comparing the physiological rates (Table 3) to the “maximal” achievable rates (Figure 3), it appears that liver, small intestine, heart, and spleen can boost their flux through the deamidated route from 15 to 40 times, while kidney and brain up to 100 times, just relying on an adequate rise of the mononucleotide substrates levels. In blood, in particular, the deamidated route peculiarly shows the capability to even overtake the amidated route, provided enough deamidated NAD precursors are supplemented [34]. Remarkably, boosting the deamidated route is a matter of increasing interest in the course of therapies involving systemic blockade of target enzymes in the amidated pathway for NAD biosynthesis, *e.g.* by FK866 or similar drugs, whereby co-supplementation of deamidated NAD precursors is claimed as a useful pharmacological support of a “block-booster” kind. In this regard, apart from unpleasant side-effects reported for high-dose Na or Qa systemic supplementation [7], [64], an effective usage of deamidated NAD precursors appears feasible in most tissues. Besides, in tissues lacking NADS, additional evaluation of such a co-supplementation strategy is warranted.

Finally, one intriguing clue arising from our metabolic reconstruction is about the NADS-catalyzed step in lung and skeletal muscle, that was found to be particularly limiting and thus determining relatively high steady-state levels of both NaMN and NaAD. Both these intermediates were reciprocally balanced by the locally abundant NMNAT activity. The observed accumulation of such deamidated intermediates was unpredicted, though indirectly described more than 40 years ago [33]. This early report, based on intraperitoneal administration of radiolabeled vitamin B3 precursors to mice, and subsequent evaluation of the radioactive NAD intermediates formed in a time-dependent fashion, provided evidence that NAD synthesis through NaAD in skeletal muscle is almost absent, and that the radioactivity accumulated as NaAD in this tissue is lost more rapidly than in other tissues. Intriguingly, though the form in which radioactivity exited was not clarified, the Authors suggested that a novel, unknown metabolite could be formed from NaAD [33]. By inspecting our data in Figure 6, it can be noted that, in lung and skeletal muscle tissues, the *in vivo* rates of the two consecutive steps within the deamidated route are not equal, being the upstream step much faster than the downstream one. Conversely, in all other tissues, the calculated rates of such consecutive steps are substantially matching, as expected for an unbranched sequence of consecutive reactions. Indeed this discrepancy is consistent with the occurrence of an alternative fate for NaAD in these tissues, other than its amidation to NAD, resulting in a putative branching point diverging from the linear pathway. The rate of this hypothetical branching reaction(s) might account for the observed difference between the upstream and downstream estimates of the deamidated route. This hypothesis is in keeping with the aforementioned report [33], and is currently under investigation in our laboratory.
